# *SynchWeb*: a modern interface for *ISPyB*

**DOI:** 10.1107/S1600576715004847

**Published:** 2015-04-25

**Authors:** S. J. Fisher, K. E. Levik, M. A. Williams, A. W. Ashton, K. E. McAuley

**Affiliations:** a Diamond Light Source, Harwell Science and Innovation Campus, Didcot, Oxfordshire OX11 0DE, UK

**Keywords:** data collection, remote access, macromolecular crystallography

## Abstract

*SynchWeb* is a modern interface to the *ISPyB* database. It significantly simplifies sample registration and is targeted towards live data collection monitoring and remote access for macromolecular crystallography. It adds a variety of new features including project management, an integrated diffraction image viewer, and a map and model viewer, as well as displaying results from automated analysis pipelines. Virtually all aspects of an experiment can be monitored through the web browser and the success of each experiment can be evaluated.

## Abstract 2

*SynchWeb* significantly simplifies sample registration and is targeted towards live data collection monitoring and remote access for macromolecular crystallography.

## 1. Introduction   

*ISPyB* (Delagenière *et al.*, 2011 ▶) is a laboratory information management system (LIMS) initially developed as a collaboration between the UK through e-HTPX (Allan *et al.*, 2005 ▶) and the European Synchrotron Radiation Facility (ESRF), and further developed in collaboration with Diamond Light Source (DLS). It was designed to deal with the large amount of information that needs to be recorded at such high-throughput synchrotron facilities. For users collecting data from many samples each hour, keeping track of data collection parameters, sample details and processing results can be a laborious task. *ISPyB* has revolutionized this by recording all the information automatically into an enterprise standard database system as the user collects each data set.

The original interface to *ISPyB* was developed in 2006. Key factors driving the development of a new interface have been the need to improve existing workflows and make the presentation of harvested data more efficient and more interactive in that users can port data to other relevant applications, such as an interactive diffraction viewer and embedded map viewer. With the increasing use of automated pipelines (Winter & McAuley, 2011 ▶) allowing for near real time data analysis, presentation of additional results has been required. The newly developed interface is targeted towards live data collection monitoring and remote access, as well as providing a variety of other useful tools for users and facility staff. In addition to the development of new features, this has also provided an opportunity to take advantage of more modern web technologies that allow the development of fast and responsive interactive interfaces.

The new interface, *SynchWeb*, was made available to the Diamond user community in March 2014. Around 99% of our users now make use of this interface. This equates to roughly 800–1000 logins per week.

## 2. Implementation   

*SynchWeb* is developed in the popular web development language PHP (PHP, 2014 ▶). The front-end uses a combination of HTML5, CSS3 and JavaScript to provide a full and responsive user interface. Access is controlled through standard central authentication system (CAS) login mechanisms. A minimal object-orientated framework was developed to allow for rapid prototyping of pages. Data are retrieved asynchronously *via* JavaScript Object Notation (JSON), making it simple to process on the client side and very lightweight with regards to bandwidth requirements. A large amount of the processing is offloaded to the client side, thus making the interface very responsive. Links are ‘rewritten’ to provide human-readable addresses that are easy to exchange with other users. *SynchWeb* is designed to work on all modern browsers including *Chrome*, *Firefox* and *Safari. SynchWeb* is released under the Apache 2 license.

### 2.1. Overview of design and functionality   

As smart phones and tablets have increased in popularity, an ever increasing group of people are using their mobile devices to browse the internet. As such, an emerging challenge for developers is supporting these devices using a single design. This has led to so-called ‘responsive design’, developing web sites that respond to the available screen space. *SynchWeb* uses such a design paradigm for the majority of its pages. A variety of the pages have a significantly different layout on mobile devices in order to make appropriate use of the available screen space. Large tables are reflowed into vertical layouts, month view calendars are collapsed into a single list and some data collection information is reformatted. With its broad functionality, described below, *SynchWeb* complements *SynchLink* (Ginn *et al.*, 2014 ▶), the iOS app developed by DLS, which is primarily orientated towards tracking data collections and viewing analysis results and presents this information very efficiently for the device it is targeted towards.

*SynchWeb* provides a fast and efficient interface guiding users all the way from sample preparation and shipping, through to experiment design and optimization, and finally experiment evaluation and structure solution. *SynchWeb* includes all of the functionality of the original *ISPyB* interface: allowing users to search proposals and visits, and register shipments, containers and samples, as well as contact details. In the new implementation most pages aim to be self-explanatory, with embedded help and visual hints. A number of pages have had significant redesigns: key improvements include reworking of the sample registration workflow, making it significantly easier to register samples. The new projects workflow (see §[Sec sec2.3]2.3) allows users to easily organize data collections and samples into a single location. The data collections page has been reworked to include all types of data collections and robot actions. The new interface adds an integrated diffraction image viewer with features similar to *Adxv* (Arvai, 2012 ▶) but accessible directly within the browser. Remote users can directly monitor the beamline through embedded webcams, and key machine and beamline parameters and their status are clearly displayed. The interface has also been extended to include results from DLS’s downstream processing pipelines such as experimental phasing, difference map generation and automated molecular replacement. Importantly, these results can be directly inspected in the browser using an integrated map and model viewer. For laboratory managers and facility staff *SynchWeb* also provides statistics for proposals and visits, giving the breakdown of time used, number of samples evaluated and any issues that occurred.

### 2.2. Sample registration and allocation   

All samples in a container can now be registered simultaneously from a single page (see supplementary material, Fig. 1). This follows the same layout as is present in the beamline control software (*GDA* at DLS). Proteins can be automatically created from this page and samples can be cloned to speed up filling containers. For protein registration users can now provide a sequence and PDB file (Protein Data Bank; Berman *et al.*, 2000 ▶) which will be automatically used for DLS’s difference map and molecular replacement pipelines (see §2.4.2).

**Figure 1:**
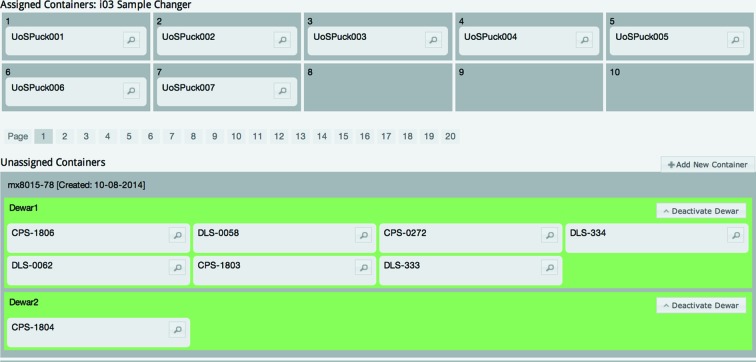
Sample allocation. Containers can be easily allocated to the beamline through a simple drag and drop interface.

Once registered, containers can be allocated to the beamline sample changer through a simple drag and drop interface (see Fig. 1 ▶). Containers can also be easily allocated to positions in the beamline sample changer through this simple drag and drop interface. Users simply click ‘Refresh’ in the beamline control software to synchronize this information. If users have registered their samples in containers prior to coming to site this significantly speeds up data collection, as sample parameters can be used in the beamline control software as variables in filenames.

#### 2.2.1. Touchscreen implementation   

As well as allocating and registering samples from a desktop computer, *SynchWeb* also provides a touchscreen interface for use in the beamline experimental hutch (see supplementary material, Fig. 2). This allows users (and facility staff handling remote users’ containers) to easily allocate containers to specific positions in the sample changer as they are physically loading samples. All relevant beamlines at DLS are equipped with touchscreen PCs in the experimental hutches.

### 2.3. Projects   

*SynchWeb* implements a project workflow that allows users to group together proteins, samples and data collections into a single location (see supplementary material, Fig. 3). This is especially useful for block allocation groups (BAGs), which often involve not only multiple research groups but also multiple universities. When users often collect hundreds of data sets per visit it can become difficult to find information from data sets that actually contain good data or keep track of a particular project. Projects can be shared with other users. Data collections, proteins and samples can all be added to a project where the book icon is displayed. Furthermore, if a protein is added to a project, all subsequent samples and associated data collections are automatically displayed in the project view.

### 2.4. Data collection   

The data collection page provides a general overview of the current visit, listing all actions in chronological order. It provides a large set of new features compared with the original *ISPyB* project. This has primarily been driven by the need to present users with results from automatic integration and structure solution pipelines, as well as adding new tools for remote data collection monitoring.

This page now displays all actions for the current visit (or ‘experiment’), which includes screenings, data collections, absorption edge scans and fluorescence spectra, as well as crystal washes and anneals. If the visit is ongoing this page will update in real time without the need to be refreshed. Data are retrieved asynchronously and are polled regularly, making this an ideal way to remotely monitor an experiment. Data collections are paginated, filterable by type (*e.g.* fluorescence spectra, absorption edge scans) and easily searched. Furthermore, individual data collections can be added to a list of favourites for ease of retrieval later.

**Figure 2:**
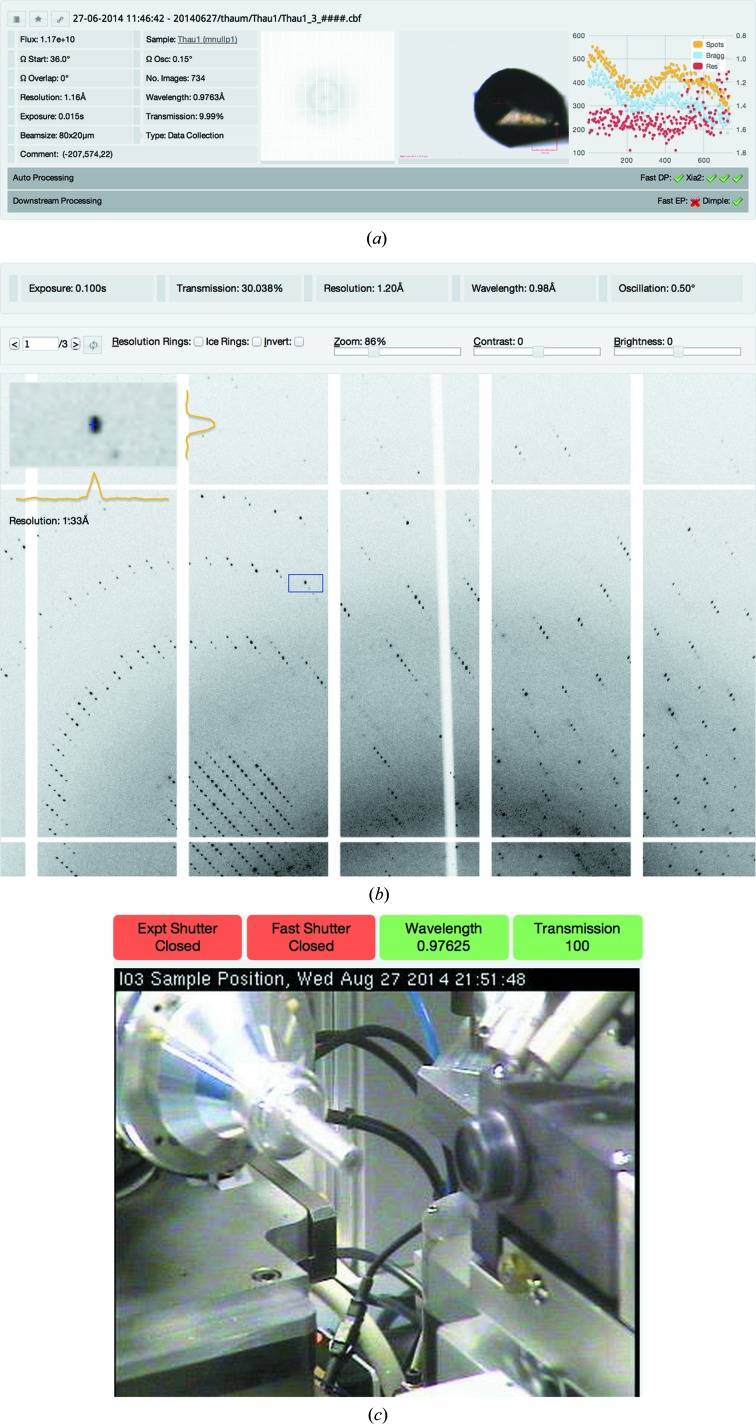
(*a*) Data collection view for a visit, showing data collection parameters, diffraction and sample thumbnails, and a *DISTL* plot. (*b*) Diffraction image viewer. (*c*) Beamline monitoring, showing webcams as well as ring and beamline status.

The page displays, for each data collection, the experimental parameters, a diffraction image thumbnail, crystal snapshots and a plot of image quality indicators as returned by *DISTL* (Zhang *et al.*, 2006 ▶) for an equally distributed number of images throughout the data collection (see Fig. 2 ▶ *a*). For screening experiments, auto-indexing results and strategies from *EDNA* (Incardona *et al.*, 2009 ▶) can be displayed, and for full data collections, auto-processing and downstream processing results can be displayed.

Clicking on a diffraction image thumbnail opens up a web-based diffraction image viewer, which is fully optimized for remote usage (see Fig. 2 ▶ *b*). The images are retrieved from the server asynchronously, and the portion of the image required is plotted onto the page. All image manipulations are then made on the client side, making the viewer very responsive. Diffraction images can be panned and zoomed, as well as corrected using brightness and contrast controls. The viewer also features a ‘zoomed’ area similar to the diffraction image viewer *Adxv*, with intensity profiles on the *X* and *Y* axes to determine spot shape. Ice and resolution rings can be appended to the image, and images can be inverted to aid the viewing of small diffraction spots. Once the first image is loaded the viewer begins caching the rest of the images of the data set, improving the response time of the viewer. This does not fully replace a dedicated diffraction image viewer as the visualization is based on a conversion of the images from their original format to a web-compatible format, but provides a very responsive way to view diffraction images remotely. As this implementation is client side, the response times for panning, zooming and other interactive features are negligible.

If the visit is ongoing then users are able to view the webcams for the beamline as well as the status of a number of synchrotron and beamline parameters, as is shown in Fig. 2 ▶(*c*). This includes data such as the wavelength and current transmission of the beamline, as well as the status of the synchrotron. The collation of this data into *SynchWeb* is extremely useful for remote users as it provides all essential information on the current status of the beamline and the storage ring in a web-based application in real time.

The results from edge scans and fluorescence spectra are depicted as shown in the supplementary material (Fig. 4). Edge scans are displayed with the resulting *CHOOCH* (Evans & Pettifer, 2001 ▶) plot if successful, along with the associated *f*′ and *f*′′ values at the peak and inflection points of the absorption edge. Fluorescence spectra are annotated with the resulting analysis from *PyMCA* (Solé *et al.*, 2007 ▶), which will try to guess the elemental content of the sample. Robot loads and unloads, as well as crystal actions such as liquid nitrogen washes and anneals, are also recorded and displayed on this page.

#### 2.4.1. Strategies and auto-processing   

**Figure 3:**
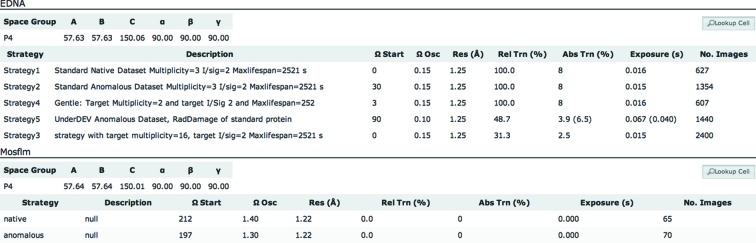
Auto-indexing results from *EDNA* and *Mosflm*. Transmission and exposure, shown in parentheses, are normalized for the maximum detector speed, *e.g.* in this case 100 Hz for PILATUS P3.

On clicking the ‘Strategies’ header, the results of auto-indexing using *EDNA* and *Mosflm* (Leslie & Powell, 2007 ▶) are displayed. Fig. 3 ▶ shows the layout of this information. Exposure times are normalized to make best use of the available flux and speed of the detector, *e.g.* 100 Hz for the PILATUS P3 6M.

**Figure 4:**
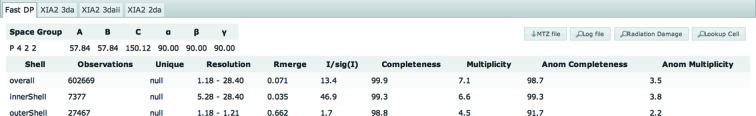
Integration pipeline results from *Fast DP* and *XIA2*. A basic radiation damage analysis is given using *XDSSTAT* plotting *R*_d_ *versus* frame number difference.

On clicking the ‘Auto Processing’ header, the results of DLS’s automatic integration pipeline using *Fast DP* (Winter & McAuley, 2011 ▶) and *XIA2* (Winter, 2010 ▶) are displayed. Fig. 4 ▶ shows the layout of this content, which includes unit-cell parameters and statistics for the overall, inner and outer shells of data. In addition, *Fast DP* now runs *XDSSTAT* (Diederichs, 2006 ▶) on all data sets, allowing for a basic analysis of radiation damage. Clicking on ‘Radiation Damage’ displays a plot of *R*_d_ *versus* frame number difference for the data set.

#### 2.4.2. Downstream processing   

**Figure 5:**
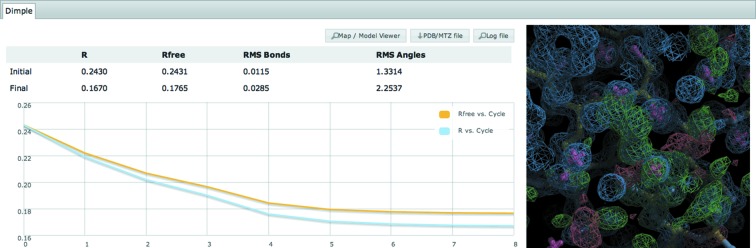
Results from *DIMPLE* showing cycle *versus R* factor and *DIMPLE* ‘blob’ images showing regions of unmodelled density. Here the bound tartaric acid in thaumatin is clearly visible.

DLS also runs a series of post integration pipelines for automated difference map generation (*DIMPLE*), molecular replacement (*AutoMrBUMP*) and experimental phasing (*Fast EP*). *DIMPLE* automatically refines the designated data set against a defined PDB file and finds unmodelled density larger than would be expected for water, thus allowing users to determine if ligands are bound a few minutes after collecting data. *AutoMrBUMP* (Keegan & Winn, 2007 ▶) uses a sequence defined for the current sample to search the PDB for models with similar sequence identity and then triggers an automatic molecular replacement pipeline. *Fast EP* automatically detects if there is a significant anomalous signal in the current data set and then uses a brute force method to search for heavy atom sites, determine the space group and hand, and ultimately phase the data set. These results can be viewed for the data collection of interest by clicking the ‘Downstream Processing’ header. For *DIMPLE* the refinement *R* factors and associated graphs are plotted, along with the *DIMPLE* ‘blob’ images showing regions of unmodelled density (see Fig. 5 ▶).

**Figure 6:**
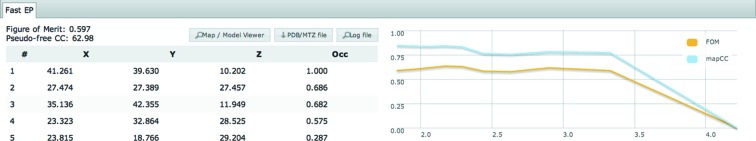
Results from *Fast EP *showing figure of merit, cc, heavy atom sites and cc/figure of merit *versus* resolution.

Results from the experimental phasing pipeline can also be viewed (see Fig. 6 ▶). This displays the determined heavy atom sites and plots of figure of merit/mapCC *versus* resolution.

#### 2.4.3. Integrated model and map viewer   

**Figure 7:**
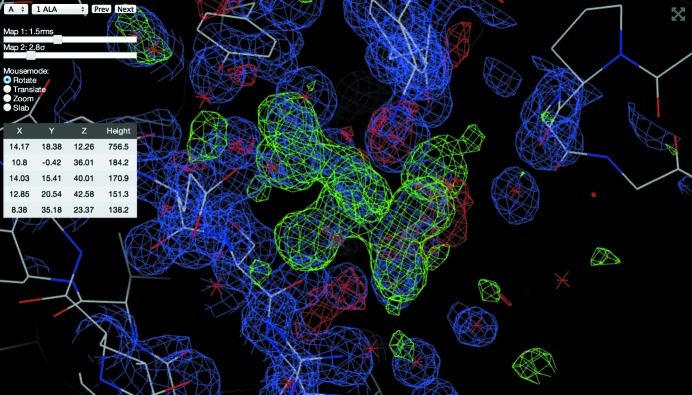
Integrated map and model viewer. Here the difference map from DIMPLE shows tartaric acid bound to thaumatin. This can also be used to view maps from the automatic phasing pipeline *Fast EP*.

In addition to displaying results from the *DIMPLE* and *Fast EP* pipelines, it is also possible to view the resulting map and models using the built-in *GLmol*-based (Nakane, 2014 ▶) viewer (see Fig. 7 ▶). This is written purely in JavaScript and makes use of WebGL. It has a similar series of hotkeys to *COOT* (Emsley & Cowtan, 2004 ▶), so *COOT* users should find it intuitive to use. Maps are compressed to reduce bandwidth requirements. Once the model and maps are loaded all modifications take place on the client side, allowing for a fast and responsive interface. With future updates of iOS (8 onwards) this should be compatible with Apple mobile devices.

### 2.5. Unit-cell search   

**Figure 8:**
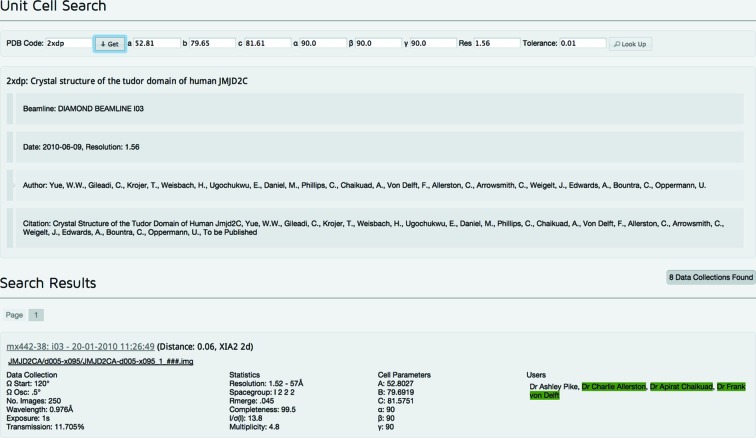
Unit-cell search, here showing auto-processing results related to PDB entry 2xdp. Authors registered on the visit that match the PDB file are highlighted in green.

One of the features in *SynchWeb* is that it allows users to trace and recover information on data collections based on a matching search of the unit-cell parameters, input directly or referred to in a deposited PDB file. These parameters are compared with those recorded in the *ISPyB* database as part of the auto-processing stage (see Fig. 8 ▶). The results are ordered by a ‘Distance’ from the searched parameters (according to the sum of the square of the differences square rooted): a larger ‘Distance’ is further from the searched parameters and a ‘Distance’ of zero is identical to the searched parameters.

Finished models are sometimes deposited with the PDB years after data were collected. Users can take the unit-cell parameters from such a model and use this new search feature to determine when, and on which beamline, the data were originally collected. This information is required for model submission and is sometimes incorrectly reported. Analysing all of the deposited model files assigned to DLS in the PDB indicates that roughly 70% of them can be identified with auto-processing results.

### 2.6. Visit statistics   

*SynchWeb* provides, for the first time, tools to allow both staff and users to evaluate how successful their beamtime was. This is especially useful for laboratory managers and BAG organizers, who need to understand how their allocated time is being used. Statistics are calculated for each visit, showing what percentage of time was used for different types of data collections, how much time the robot used, and how much time was remaining at the end of the visit. The average number of data collections and samples loaded per hour are also displayed. This page also provides the date, time and duration of any issues that occurred during the experiment. In the case of beamline faults, a full report can be accessed if this has been recorded by the facility staff.

## 3. Conclusions   

As the productivity of macromolecular crystallography beamlines continues to increase, thanks to advancements in instrumentation [faster detectors (Broennimann *et al.*, 2006 ▶), improved sample changers and automation pipelines], the amount of information that needs to be stored continues to soar. *SynchWeb* provides a modern interface to the *ISPyB* database, allowing users to manage metadata and processing results associated with their data collections. Users are easily able to provide sample information before an experiment commences by creating shipments and samples, and then monitor the status of experiments in progress, and analyse collected data post-visit. The system is optimized for remote use, where traditional remote desktop applications are typically very unresponsive. Indeed, significant effort has been made to achieve a responsive design, allowing users and staff to monitor experiments *via* a variety of devices. *SynchWeb* provides a wealth of new features compared with the previous interface, aiding both users and staff members in managing the beamline and beamtime.

Future developments will focus on features to manage *in situ* sample registration and data collections, such as plate registration and pipelines for automatically integrating and solving such data sets. We also intend to make use of *SynchWeb* in other disciplines.

*SynchWeb* can be accessed from http://ispyb.diamond.ac.uk if you are a registered user of DLS.

## Supplementary Material

Supplementary figures. DOI: 10.1107/S1600576715004847/fs5101sup1.pdf
