# Supplementary material for: SynchWeb: a modern interface for ISPyB
Source: J Appl Crystallogr. 2015 Apr 25;48(Pt 3):927–32. doi: 10.1107/S1600576715004847 (PMC4453979; doi:10.1107/S1600576715004847)
Supplement: Supplementary file 1 [file j-48-00927-sup1.pdf]

SynchWeb – A modern interface for ISPyB: Supplementary Material

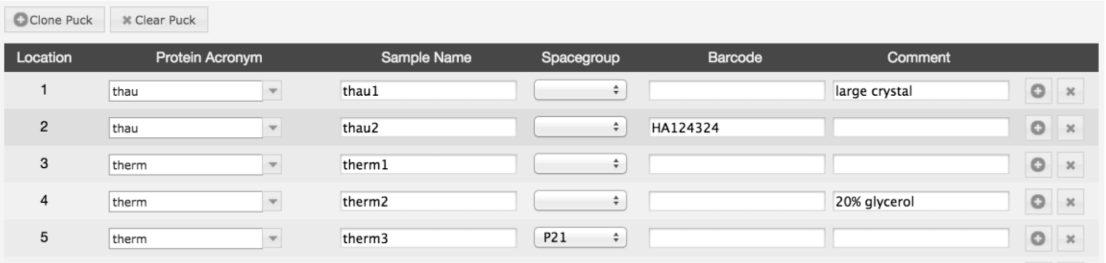

Figure 1: Sample registration. This is closely modelled on the beamline control software; samples can be easily cloned to speed up filling containers.

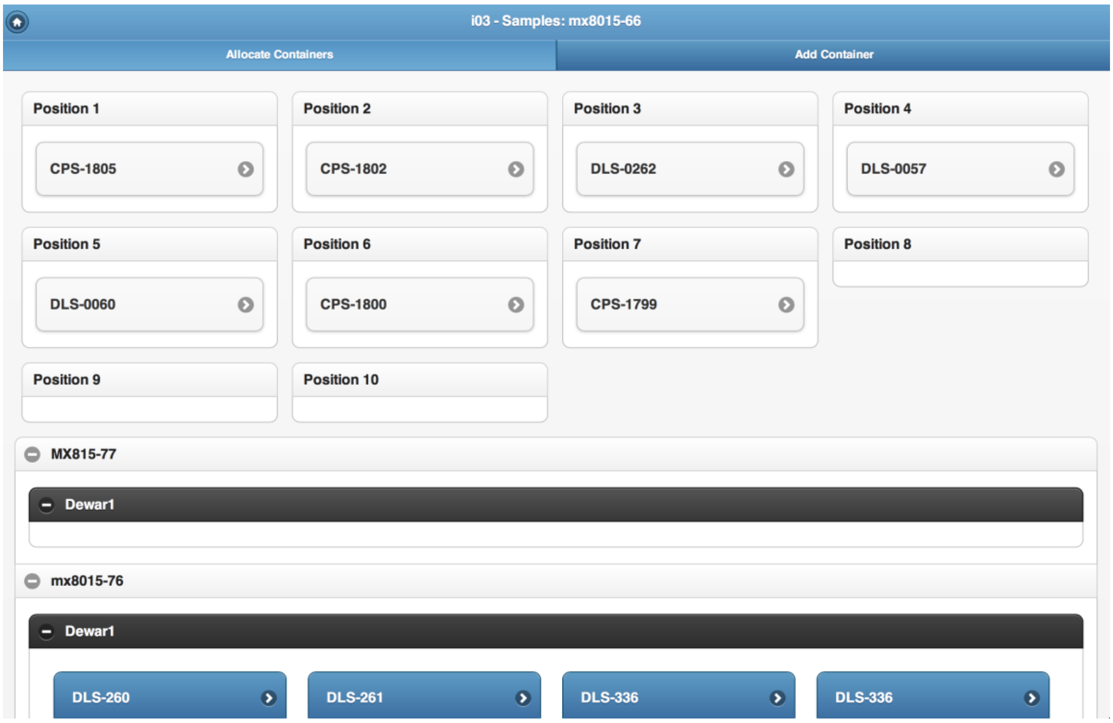

Figure 2: Touchscreen sample allocation. This allows users to allocate samples to the beamline control software directly in the experimental hutch as they are physically loading pucks.

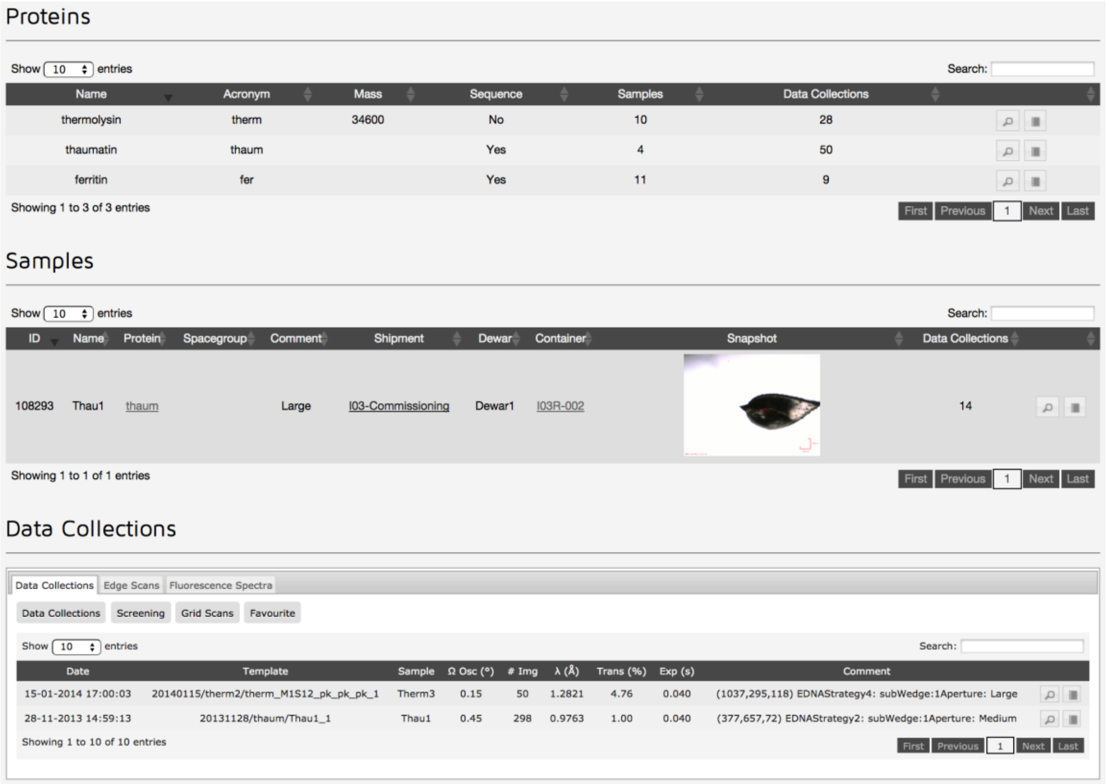

Figure 3: Project organisation, allowing users to group proteins, samples, and data collections together into a common place that can be shared with other users.

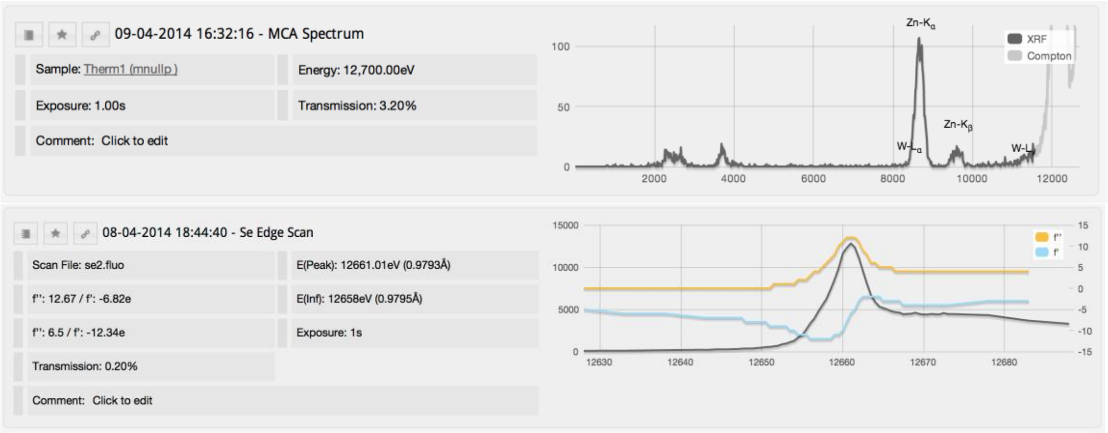

Figure 4: MCA fluorescence spectrum with analysis from PyMCA (top), and edge scan with associated CHOOCH plot (bottom)
